# Cross detection for odor of metabolic waste between breast and colorectal cancer using canine olfaction

**DOI:** 10.1371/journal.pone.0192629

**Published:** 2018-02-13

**Authors:** In-Seok Seo, Hwan-Gon Lee, Bonkon Koo, Chin Su Koh, Hae-Yong Park, Changkyun Im, Hyung-Cheul Shin

**Affiliations:** 1 College of Medicine, Hallym University, Chuncheon, Korea; 2 Gangwon Provincial Police Agency, Chuncheon, Korea; 3 Department of Physical Education, Hallym University, Chuncheon, Korea; 4 School of Interdisciplinary Bioscience and Bioengineering, POSTECH, Pohang, Korea; Duke University, UNITED STATES

## Abstract

Although several studies have been performed to detect cancer using canine olfaction, none have investigated whether canine olfaction trained to the specific odor of one cancer is able to detect odor related to other unfamiliar cancers. To resolve this issue, we employed breast and colorectal cancer in vitro, and investigated whether trained dogs to odor related to metabolic waste from breast cancer are able to detect it from colorectal cancer, and vice versa. The culture liquid samples used in the cultivation of cancerous cells (4T1 and CT26) were employed as an experimental group. Two different breeds of dogs were trained for the different cancer odor each other. The dogs were then tested using a double-blind method and cross-test to determine whether they could correctly detect the experimental group, which contains the specific odor for metabolic waste of familiar or unfamiliar cancer. For two cancers, both dogs regardless of whether training or non-training showed that accuracy was over 90%, and sensitivity and specificity were over 0.9, respectively. Through these results, it was verified that the superior olfactory ability of dogs can discriminate odor for metabolic waste of cancer cells from it of benign cells, and that the specific odor for metabolic waste of breast cancer has not significant differences to it of colorectal cancer. That is, it testifies that metabolic waste between breast and colorectal cancer have the common specific odor in vitro. Accordingly, a trained dogs for detecting odor for metabolic waste of breast cancer can perceive it of colorectal cancer, and vice versa. In order to the future work, we will plan in vivo experiment for the two cancers and suggest research as to what kind of cancers have the common specific odor. Furthermore, the relationship between breast and colorectal cancer should be investigated using other research methods.

## Introduction

Non-invasive cancer diagnostic techniques such as CT, MRI, PET, ultrasound, serum biomarker tests, etc. have been developed using state-of-the-art biomedical engineering technology. Although these instruments show relatively high accuracy for cancer diagnosis, there still remain drawbacks such as high cost, long delays required for diagnosis, and the induction of some health risks (e.g., an increase in the risk of cancer by radiation exposure, and the danger of unnecessary biopsies) [1]. The possibility of a novel cancer diagnostic method using the superior canine olfactory ability was first reported by [2]. Since then, many studies on cancer diagnosis with canine scent detection have been reported. A detection dog, as employed in these studies, is defined as a dog that is ethologically trained to identify specific chemical odors such as explosives and drugs, and to notify the handler or a third person through specific actions. Researches related to cancer diagnosis using detection dogs have been conducted for various kinds of cancer (e.g., bladder [3], breast [4, 5], colorectal [6], lung [4], melanoma [2, 7], ovarian [8, 9], and prostate cancers [5, 10–12], and using diverse odor samples (e.g., breath, urine, stool, malignant tumor tissue, blood samples) from humans. Most of these studies have focused on the feasibility and performance of cancer detection using canine olfaction for not several cancers but just one cancer. That is, it has been just verified whether a detection dog trained for an odor sample of one cancer is able to detect another odor samples of the same cancer.

The research by [8], which showed 100% sensitivity and 97.5% specificity by canine scent detection using ovarian carcinoma samples, suggested that the odor detection of ovarian carcinoma is due to specific odor. [6] compared consequence of diagnosis for exhaled breath and watery stool samples of patients with colorectal cancer by canine scent detection with result of diagnosis by colonoscopy, and suggested that cancer emit a specific volatile odor, and chemical compounds emitting specific odor circulate through the body. Metabolic waste, which is excreted during the course of human metabolism, are eliminated from the human body through breath, blood, saliva, skin, stool and urine [13]. Because the metabolism of cancerous cells differs from it of benign cell, the metabolic waste generated by cancerous cells can produce the specific volatile odors dissimilar to it generated by benign cells [14, 15]. [16] investigated air samples from the headspace of the culture vessel from the culture of CALU-1, which is a non-small-cell lung cancer cell line, and revealed the existence of certain volatile compounds derived from the cancer cells. Accordingly, it can be assumed that odor samples, such as exhaled breath, urine and stool, from human used in studies of cancer detection by canine olfaction contain metabolic waste related to cancer, and that dogs detect these characteristic volatile odor related to the metabolic waste produced by cancer cell metabolism.

At this point, we raise a question as to whether detection dogs trained for a specific volatile odor produced by metabolic waste of one cancer can detect it of other unfamiliar cancer. In order to explore the clue to this question, we developed the following hypotheses: 1. If canine olfaction can differentiate odor related to metabolic waste of cancer cells from it of benign cells, specific volatile odors related to cancer detection using canine olfaction must be originating from metabolic waste of cancer cell. 2. If hypothesis 1 is verified as true, canine olfaction trained for odor of just one cancer will not be able to detect it of other cancers. To confirm the above hypotheses, we employed culture liquid samples used to cultivate two cancerous cells (breast cancer and colorectal cancer, which is a completely different type) as odor sample by metabolic waste, two dogs of a different breed, and cross-test method. Therefore, the objective of this study is to identify whether canine olfaction is able to differentiate the specific odor from the metabolic waste of one cancer from it from other unfamiliar cancer.

## Materials and methods

### Experimental group (training and test samples)

The cancerous cells cultivated in this study were 4T1 (mammary carcinoma cells) and CT26 (colorectal carcinoma cells) using mouse cells purchased from ATCC. These cancerous cells were cultivated in cell culture dishes (100 mm) filled with DMEM (Dulbecco modified Eagle’s medium) containing 10% FBS (fetal bovine serum) and 1% penicillin/streptomycin (Invitrogen, Grand Island, NY), at 5% CO_2_, 95% humidity and 37°C for 4 days.

### Samples for control group

The control group consisted of four samples such as two culture liquid samples used in the cultivation of primary cell isolated from mammary and colorectal tissue of noncancerous female mice, one culture liquid sample unused for culture except the cancer cells and primary cells and one empty sample. To rule out possibility that the detection dogs would become aware of the excipients, all the culture liquid used in the cancer cell and primary cell culture were contained exactly identical components, and cultured under the same culture conditions (temperature and humidity) as the cancerous cells for 4 days.

### Dogs

An untrained Cocker Spaniel (2 years, male, Dog-1) and English Springer Spaniel (2 years, male, Dog-2) were selected as detection dogs for this study. The source of the dogs were the korea police dog training center, and the dogs were housed in kennels at hallym university. The dogs were trained to the basal detection using odor of training samples of each different cancer, with 2 training sessions/day (30 minutes/session) for six months conducted by an explosive detection dog trainer from the national police in Korea. During the training period, the dogs were maintained in a constant temperature (22±2°C) and humidity (55±5%) facility, and were provided with feed twice per day.

### Training

**Step 1**. Through reward training by vision using dummies, the ability of the dogs to respond to specific odors was gradually enhanced.
**Step 2**. Through reward training by olfaction, when the dog correctly identified the target, i.e. the dummy, located in one of five boxes, the”sit” command was given directly to the dog in front of the dummy, and the dog received the dummy as a reward. This step was repeated until the dog reflexively sat in front of the target after detection.
**Step 3**. In this course, both training sample from the experimental group and the dummy were placed in the same box. If the target box out of five boxes was correctly detected by the dog, the dummy was given to the dog with a tug-of-war game accompanied with praise. Through this process, the dogs were adapted to the odor of training samples.
**Step 4**. The same process as step 3 was repeated using just the training samples from the experimental group, without the dummy.

### Experimental setup

The boxes (350×150×170 mm) used in the study were made with acrylic plastic, and five boxes were used for training and testing. A hole (65 mm in diameter) which is filled with either the experimental or control group was placed in the center of the boxes. To protect the odor samples(experimental and control group) from contamination, all the samples were placed in separate 5 ml microtubes and kept in a freezer at -70°C. The frozen microtubes were thawed at room temperature (22–25°C) 2 hours before the training or test, and the culture liquids in the microtubes were poured into glass jars (55 mm in diameter, 85 mm in height). During the training and test, five boxes were arranged in a line. One sample from the experimental group and four samples from the control group were placed randomly in the boxes using computer program. All the samples used in the experiment were used only once.

### Tests

Five samples (one experimental sample and four control samples) per trial have been tested by one detection dog, and 20 trials per day for one detection dog were performed for 8 days using a double-blind method and cross-test. Both the handler and the judge were unaware of the position for samples of the experimental and control group. One experimental sample and four controls were arranged randomly in five boxes by a third person before the test. The total number of odor samples from the experimental and control group used in the test was 320 and 1,280, respectively.

#### Cross-test

The test divided into two stages. In the 1^st^ stage, it performed the test using the familiar training samples for 4 days, and then achieved the test employing the unfamiliar cancer samples except of the training samples for other 4 days under the 2^nd^ stage.

### Statistical methods

To evaluate the performance for the detection of test samples by canine olfaction, the detection accuracy, sensitivity and specificity were calculated using the MATLAB program (The MathWorks, Inc., USA). Also, to identify the statistical differences for the detection results between the two dogs and the two cancers, statistical significance (*p*-value) was computed with an independent sample t-test.

### Ethical approval

This study was approved by the Institutional Animal Care and Use Committee (IACUC) at Hallym University in Korea.

## Results

### The detection accuracy by the two dogs

The detection results for each of the training samples over 4 days are shown in Table 1. The detection accuracy for the 4T1 training samples was 94% (75/80) by Dog-1, and the detection accuracy for the CT26 training samples was 94% (75/80) by Dog-2. The detection results for each of the test samples from the unfamiliar cancers over 4 days are shown in Table 2. The detection accuracy for the CT26 test samples by Dog-1 was 91% (73/80), and the detection accuracy for the 4T1 test samples by Dog-2 was 95% (76/80).

**Table 1:** Detection accuracy for the familiar cancer samples.

Day	Dog-1(4T1)	Dog-2(CT26)
1^st^	18/20 (90%)	17/20 (85%)
2^nd^	19/20 (95%)	19/20 (95%)
3^rd^	19/20 (95%)	19/20 (95%)
4^th^	19/20 (95%)	20/20 (100%)
Sum	75/80 (94%)	75/80 (94%)
S.D.	0.025	0.063

**Table 2:** Detection accuracy for the unfamiliar cancer samples.

Day	Dog-1(CT26)	Dog-2(4T1)
1^st^	18/20 (90%)	19/20 (95%)
2^nd^	18/20 (90%)	20/20 (100%)
3^rd^	19/20 (95%)	18/20 (90%)
4^th^	18/20 (90%)	19/20 (90%)
Sum	73/80 (91%)	76/80 (95%)
S.D.	0.025	0.041

### The sensitivity and specificity by the two dogs

The sensitivity and specificity for the training and test samples by two dogs over 8 days are shown in Tables 3 and 4, respectively. The mean sensitivity by the two dogs was 0.944 for 4T1, and 0.926 for CT26. The mean specificity by the two dogs was 0.986 for 4T1, and 0.981 for CT26. [4] reported 0.88 sensitivity and 0.98 specificity using exhaled breath samples from humans for the detection of breast cancer by canine olfaction. [6] evaluated the detection accuracy of colorectal cancer using exhaled breath and watery stool samples from human by canine scent detection, and showed that the sensitivity for breath samples was 0.91 and the specificity was 0.99. The sensitivity for stool samples was 0.97 and the specificity was 0.99. The detection results for these studies correspond with our study on breast cancer and colorectal cancer, although the number of subjects and dogs, and the kind of odor samples were different.

**Table 3:** Sensitivity by the two dogs.

Sensitivity	4T1	CT26	Mean
Dog-1	0.938	0.913	0.926
Dog-2	0.950	0.938	0.944
Mean	0.944	0.926	0.935

**Table 4:** Specificity by the two dogs.

Specificity	4T1	CT26	Mean
Dog-1	0.984	0.978	0.981
Dog-2	0.988	0.984	0.986
Mean	0.986	0.981	0.984

### The statistical differences between the two dogs and the two cancers

**Fig 1:**
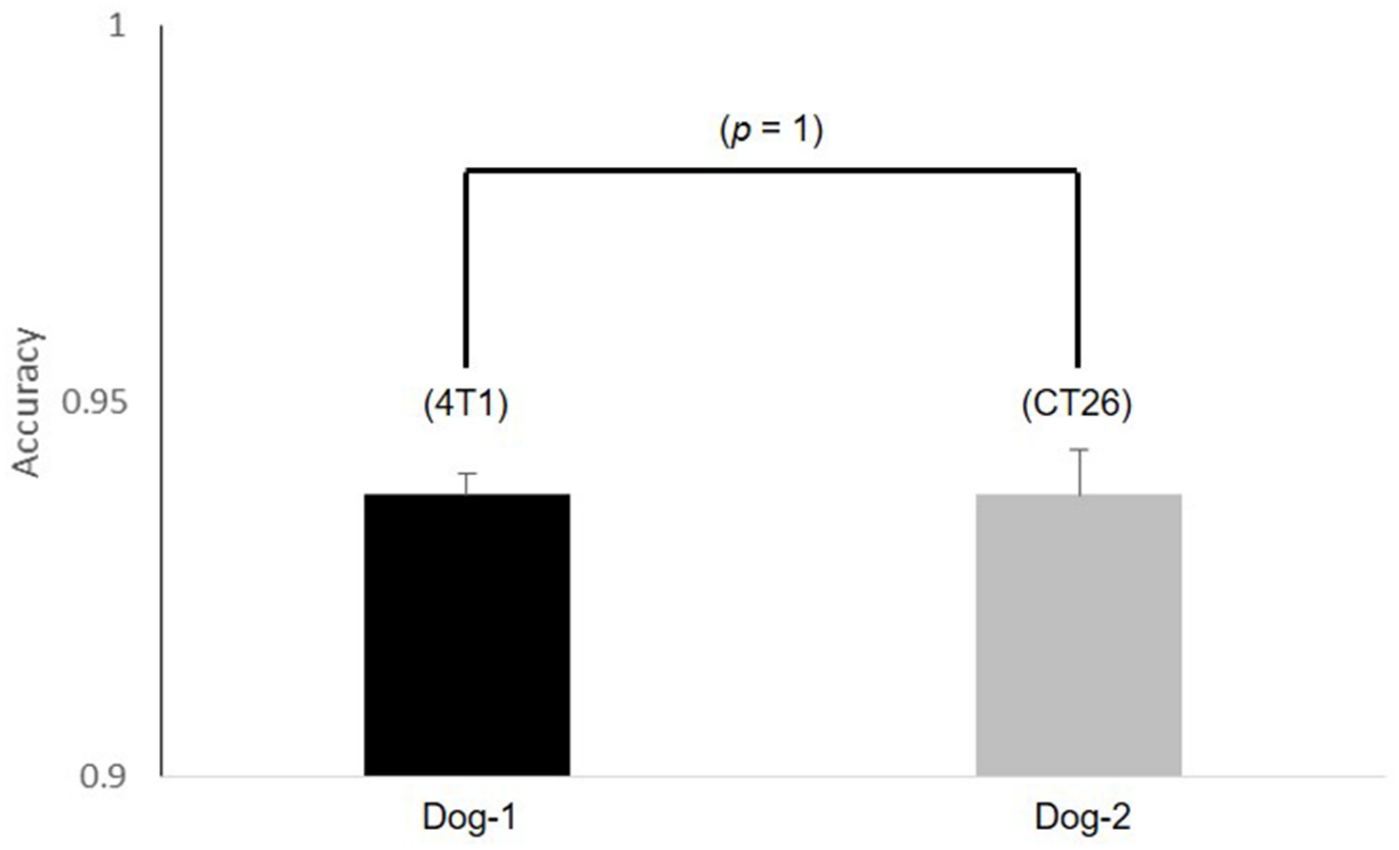
The statistical differences between two dogs for each training sample.

**Fig 2:**
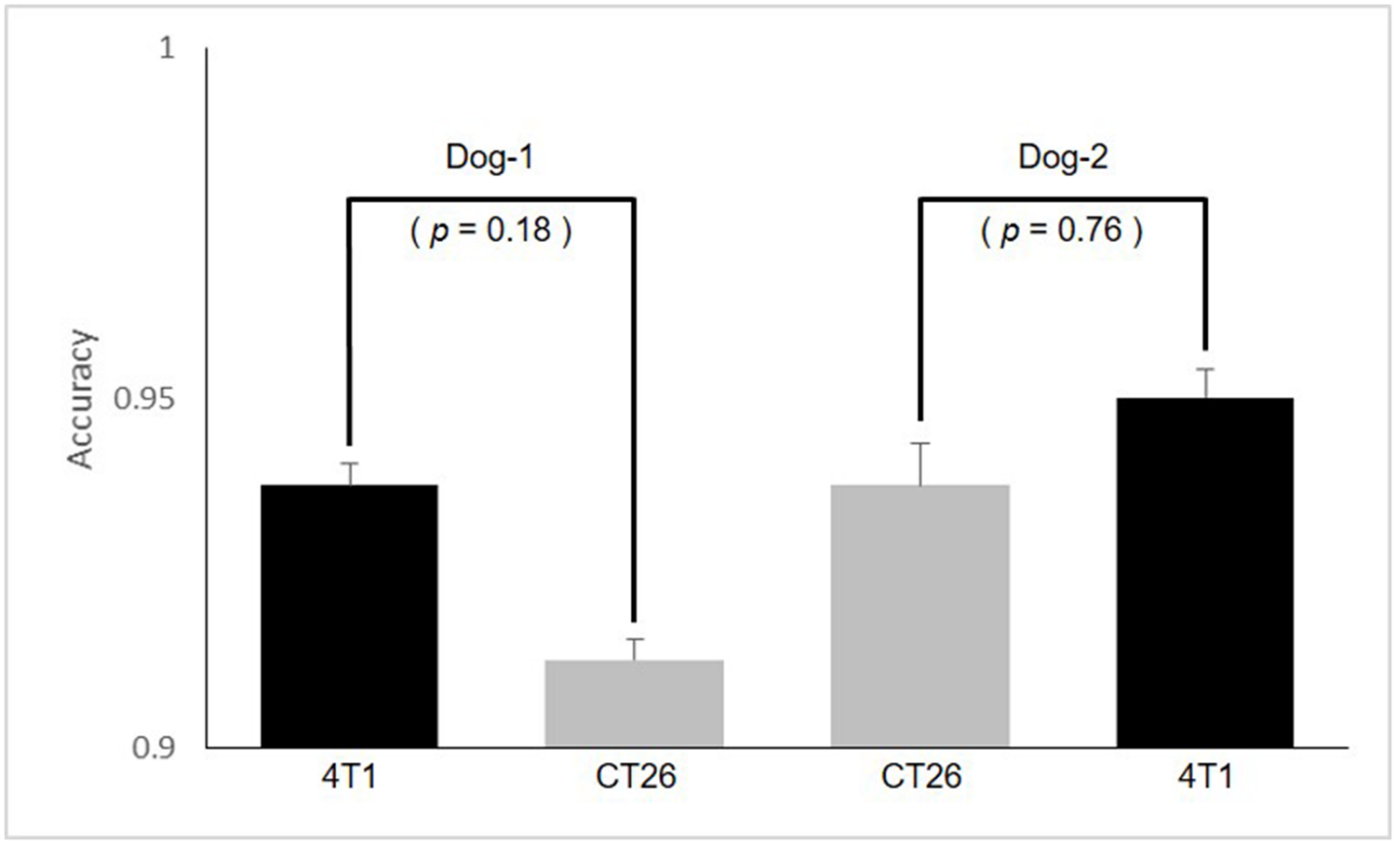
The statistical differences between two cancer samples for each dog.

There were no statistical differences in detection accuracy between the two dogs for the familiar training samples (Fig 1). That is, the two dogs could detect the odor of the trained cancer samples with accuracy over 90% regardless of the dog breed. There were also no statistical differences in detection accuracy between the two cancers for each dog (Fig 2). Although odor of one out of the two cancers did not trained to the dogs, the odor related to metabolic waste of the breast and colorectal cancer could be detected with accuracy over 90% regardless of the kind of two cancers by detection dogs. That is, the two dogs trained to the odor of just one cancer out of the both cancers were possible to detect the breast and colorectal cancer regardless of whether training or non-training. This result means that metabolic waste between the both cancers are possessed of the common specific odor each other.

## Discussion

This study was performed to identify whether the superior canine olfactory ability of dogs can distinguish the other cancer odor except trained cancer odor relating to breast and colorectal cancer. Through this study, we get the findings that a specific odor from metabolic waste of cancer cells is different to it from metabolic waste of benign cells, and this result proves that hypothesis 1 is true. That is, the odor of detected cancer by dogs is originated from the metabolic waste of cancer. But, other result of this study proves that hypothesis 2 is false. That is, trained dog for odor of metabolic waste of breast cancer was able to detect it of colorectal cancer, and vice versa. This means that breast and colorectal cancer have the common specific volatile odor each other, and demonstrates the similarity of metabolic waste between the both cancers.

Breast and colorectal cancer are the most prevalent cancers in women in the developed countries. Because it has been continually reported that an attack of one out of the both cancers is prone to increase incidence of the other in the same women, the probability for the correlation between breast and colorectal cancer has been constantly raised for a long time, and in order to investigate the relationship between breast and colorectal cancer, various researches have been performed in an aspect of etiology, epidemiology, genetics, family history, environmental factors etc. The results of these researches have been ambiguous and controversial. In etiological researches for this issue, it has been reported that breast and colorectal cancer were correlated with some dietary factors [17] and socio-economic factors [18]. Also, several epidemiological researches have suggested that an attack of one out of the both cancers may affect with the development of the other [19], but [20] reported that breast cancer did not increase the risk of colorectal cancer in women through research using the Surveillance Epidemiology and End Results(SEER) database. From researches with the viewpoint of family history, the existence of familial breast and colorectal cancer is equivocal [21]. The Cancer Genome Atlas (TCGA) project [22] have analyzed various cancers(including breast and colorectal cancer) for the molecular level, but it is not still revealed whether breast cancer correlates with colorectal cancer. However, from the results of this study, it is discovered to exist the common specific odor between metabolic waste of breast and colorectal cancer using canine olfaction in the viewpoint of odor. This presents evidence for the possibility which breast cancer can be correlated with colorectal cancer reciprocally.

An odor is generally caused by chemical compounds. [23] disclosed that the more complex the molecular structure of odorants, the more numerous the kinds of odors, and [24] developed machine-learning algorithms to predict odor intensity and pleasantness based on chemoinformatic features of a large olfactory psychophysical data set. Although various researches to verify the connection between molecular structure of odorant and perception have been performed, it is not still predicted what odor is provoked from molecular structure [25]. In spite of the relationship between molecular structure and odor is not clearly solved, the existence of the common specific odor between breast and colorectal cancer in the viewpoint of cancer diagnostic method using odor detection may be contributed to future work for verifying correlation between the both cancers. To the best of our knowledge, our research is the first study that identify the existence of the common specific odor for metabolic waste between breast and colorectal cancer using canine olfaction.

Through the results of this study, we present proposal such as following. Because the metabolic waste of the breast and colorectal cancer have the common specific odor reciprocally, detection dogs trained to the specific odor for metabolic waste of breast cancer is possible to detect it of colorectal cancer except of training, and vice versa. Also, this study may be evidence to testify to the relationship between breast and colorectal cancer. Furthermore, it is required investigation as to whether the common specific odor related to metabolic waste exist in other cancers besides breast and colorectal cancer.

## Conclusions

Through this experimental process, we verified that cancer detection using canine olfaction is possible due to odor from metabolic waste of cancer cell, and that the common specific odor exists between metabolic waste of breast and colorectal cancer. Accordingly, we anticipate that human cancer diagnosis harnessing the superior canine olfactory ability is a promising alternative for the diagnosis of breast and colorectal cancer. Additionally, we suggest that correlation between breast and colorectal cancer should be investigated through biochemical analysis in the future, and that the possibility for existence of the common specific odor between other cancers should be investigated using not only canine olfaction but also another analysis methods.

## Supporting information

S1 Table Detection accuracy for the test cancer samples. (PDF)
