# Supplementary material for: Cross detection for odor of metabolic waste between breast and colorectal cancer using canine olfaction
Source: PLoS One. 2018 Feb 13;13(2):e0192629. doi: 10.1371/journal.pone.0192629 (PMC5811037; doi:10.1371/journal.pone.0192629)
Supplement: S1 Table — (PDF) [file pone.0192629.s001.pdf]

Table S1A. Detection accuracy for the familiar cancer samples

Day	Dog-1(4T1)																					Dog-2(CT26)																				
	Trial																					Trial																				
1st	1	2	3	4	5	6	7	8	9	10	11	12	13	14	15	16	17	18	19	20	sum	1	2	3	4	5	6	7	8	9	10	11	12	13	14	15	16	17	18	19	20	sum
	○	○	○	○	○	○	○	○	○	○	×	○	○	○	○	○	○	×	○	○	18/20	○	○	○	○	○	×	○	○	○	×	○	○	○	○	○	○	○	×	○	○	17/20
2nd	1	2	3	4	5	6	7	8	9	10	11	12	13	14	15	16	17	18	19	20	sum	1	2	3	4	5	6	7	8	9	10	11	12	13	14	15	16	17	18	19	20	sum
	○	○	○	○	○	○	○	○	○	○	○	○	○	○	○	×	○	○	○	○	19/20	○	○	○	○	○	○	○	○	○	○	○	○	○	○	○	○	×	○	○	○	19/20
3rd	1	2	3	4	5	6	7	8	9	10	11	12	13	14	15	16	17	18	19	20	sum	1	2	3	4	5	6	7	8	9	10	11	12	13	14	15	16	17	18	19	20	sum
	○	○	○	○	○	○	○	○	×	○	○	○	○	○	○	○	○	○	○	○	19/20	○	○	○	○	○	○	○	○	○	○	○	×	○	○	○	○	○	○	○	○	19/20
4th	1	2	3	4	5	6	7	8	9	10	11	12	13	14	15	16	17	18	19	20	sum	1	2	3	4	5	6	7	8	9	10	11	12	13	14	15	16	17	18	19	20	sum
	○	○	○	○	○	○	○	○	○	○	○	○	○	○	○	○	○	○	×	○	19/20	○	○	○	○	○	○	○	○	○	○	○	○	○	○	○	○	○	○	○	○	20/20

(○: Success for the related cancer detection, ×: Failure for the related cancer detection)

Table S1B. Detection accuracy for the unfamiliar cancer samples

Day	Dog-1(CT26)																					Dog-2(4T1)																				
	Trial																					Trial																				
1st	1	2	3	4	5	6	7	8	9	10	11	12	13	14	15	16	17	18	19	20	sum	1	2	3	4	5	6	7	8	9	10	11	12	13	14	15	16	17	18	19	20	sum
	○	○	○	○	○	○	○	○	○	○	○	○	×	○	×	○	○	○	○	○	18/20	○	○	○	○	○	○	○	×	○	○	○	○	○	○	○	○	○	○	○	○	19/20
2nd	1	2	3	4	5	6	7	8	9	10	11	12	13	14	15	16	17	18	19	20	sum	1	2	3	4	5	6	7	8	9	10	11	12	13	14	15	16	17	18	19	20	sum
	○	○	○	○	○	○	○	×	○	○	○	○	○	○	○	○	×	○	○	○	18/20	○	○	○	○	○	○	○	○	○	○	○	○	○	○	○	○	○	○	○	○	20/20
3rd	1	2	3	4	5	6	7	8	9	10	11	12	13	14	15	16	17	18	19	20	sum	1	2	3	4	5	6	7	8	9	10	11	12	13	14	15	16	17	18	19	20	sum
	○	○	○	○	○	○	○	○	○	○	○	○	○	○	○	○	○	○	×	○	19/20	○	○	○	○	○	○	○	○	○	○	○	×	○	○	○	○	○	○	×	○	18/20
4th	1	2	3	4	5	6	7	8	9	10	11	12	13	14	15	16	17	18	19	20	sum	1	2	3	4	5	6	7	8	9	10	11	12	13	14	15	16	17	18	19	20	sum
	○	○	○	○	○	○	○	○	×	○	○	○	○	○	○	×	○	○	○	○	18/20	○	○	○	○	○	○	○	○	○	○	○	○	○	○	○	×	○	○	○	○	19/20

(○: Success for the related cancer detection, ×: Failure for the related cancer detection)
